# Pectin Extracted by a Recyclable Molecular Mixture: A Promising Material for Porous Membranes in Quasi-Solid-State Na-Ion Batteries

**DOI:** 10.1021/acssuschemeng.5c07306

**Published:** 2025-10-10

**Authors:** Wenli Wang, Pedro Y. S. Nakasu, Josiel Martins Costa, Francesco D’Acierno, Niyaz Ahmad, Maria Magdalena Titirici, Daniele Pontiroli, Mauro Riccò, Changwei Hu, Jason P. Hallett

**Affiliations:** † Key Laboratory of Green Chemistry and Technology, Ministry of Education, National and Local Joint Engineering Laboratory of Energy Plant Biofuel Preparation and Utilization, College of Chemistry, 12530 Sichuan University, 29 Wangjiang Road, Chengdu, Sichuan 610064, PR China; ‡ Department of Chemical Engineering, 4615 Imperial College London, London SW7 2AZ, U.K.; § Department of Chemical Engineering, 549427 KU Leuven, Gebroeders de Smetstraat 1, Ghent 9000, Belgium; ∥ Nanocarbon Laboratory, Department of Mathematical, Physical and Computer Sciences, 9370 University of Parma, Parco Area delle Scienze 7/A, Parma 43124, Italy

**Keywords:** galacturonic acid, green solvent, ionic liquids, renewable materials, energy storage materials, circular economy

## Abstract

In this work, we explored a solvent-based extraction of pectin from apple pomace and evaluated the extracted pectin as a precursor for porous quasi-solid-state Na-ion battery membranes, alongside testing hard carbon derived from commercial pectin as an electrode material. A screening of 10 distinct ionic liquids (ILs) and 6 different antisolvents revealed that *N*,*N*-dimethylbutylammonium acetate ([DMBA]­[OAc]), combined with ethanol, was highly effective in pectin extraction, while the same IL with 1-butanol as antisolvent yielded a purer form of the pectin product. Additionally, [DMBA]­[OAc] did not behave as an IL and resembled a molecular mixture based on ^1^H NMR and conductivity measurements. A two-level fractional design was employed with four factors, considering temperature, solid loading, acid-to-base ratio (ABR), and water content of ILs, where temperature and solid loading were significant factors. The optimized conditions for the pectin extraction were at 80 °C, 1 h, with 15 wt % of solids loading, an ABR of 1.1, and 30 wt % of water content (IL), which yielded 9.6 wt % of pectin. The resulting quasi-solid-state Na-ion half-cell exhibited a capacity of ∼130 mAh g^–1^ at C/20, making it an excellent candidate for energy storage applications.

## Graphical abstract

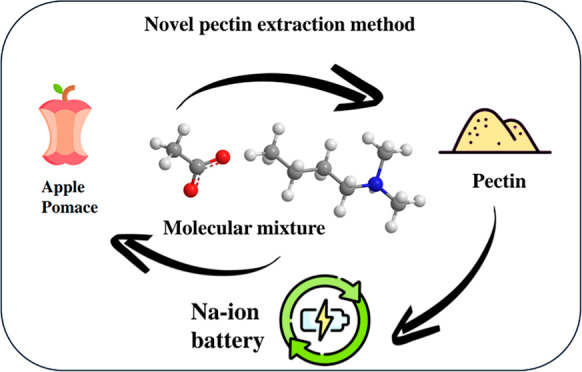

## Introduction

According to the United Nations Environment Programme, more than one billion meals are wasted globally each day, with food loss and waste accounting for 8–10% of global greenhouse gas emissions. This inefficiency occupies nearly one-third of the world’s agricultural land and contributes significantly to biodiversity loss.^1^ An estimated 35–40% of unavoidable food supply chain waste (FSCW) is generated during processing from farm to table.^2^ Due to its potential to produce biochemicals, biofuels, and functional materials, FSCW is considered an important economic biorefinery feedstock. For instance, pectin obtained from FSCW, such as apple, citrus, and grapefruit pomace via acid extraction, can be used in the healthcare, food, cosmetic, and polymer-processing industries.^3^ Consequently, the valorization of FSCW will play a significant role in mitigating food loss and waste, reducing climate impacts and economic losses, and accelerating progress toward the Sustainable Development Goals.

Apple pomace is a promising raw material for pectin extraction due to its abundance in the plant matrix. Apple is one of the most common fruits, and its global production has reached 81.6 million tons in 2022.^4^ Byproducts from apple processing account for approximately 25–30 wt % of the total fruit mass, highlighting their potential as a feedstock for biobased materials within the context of FSCW biorefineries. Pectin, a plant-derived heteropolysaccharide, is composed of 17 different monosaccharides.^5^ The backbone of pectin consists primarily of (1 → 4)-linked α-d-galacturonic acid (GalA) residues, accounting for over 70% of its structure.^6^ Based on the variability of its side chains, pectin can be categorized into several types, including homogalacturonan, rhamnogalacturonan I, rhamnogalacturonan II, xylogalacturonan, and apiogalacturonan.^7^

Pectin is classified according to the degree of esterification (DE) of its carboxylic acid groups: high methoxy pectin (HMP), in which more than 50% of the carboxyl groups are methyl-esterified, and low methoxy pectin (LMP), with a DE below 50%.^8^ The structural features of pectin strongly influence its physicochemical properties and potential applications. For example, DE affects its gelling and thickening behavior. HMP forms gels in acidic environments (pH 2–3.5) when combined with high sugar concentrations (55–75%), while LMP gels over a broader pH range (2–6) and requires only small amounts of sugar in the presence of divalent cations such as calcium (Ca^2+^).^9^ Due to its distinct properties, HMP is utilized in the food industry as a gelling agent, stabilizer, emulsifier, and thickener to produce jams and jellies. On the other hand, LMP can serve as a fat replacer in spreads, ice cream, fruit preparations for yogurt, heat-reversible bakery glazing, emulsified meat products, and low-calorie products such as diet carbonated beverages.^10^ Additionally, due to its high water-solubility, exceptional film-forming ability, great flexibility, and various other crucial properties, such as serving as a barrier to moisture, oil, and aroma, pectin can be employed either directly in food products or as an edible coating in the form of a preformed film that envelops the food.^5,11^

Researchers have explored various systems for pectin extraction from FSCW, with yields varying depending on feedstocks, extraction methods, and operational parameters. The industrial extraction of pectin typically involves inorganic and organic acids, such as HCl, H_2_SO_4_, HNO_3_, citric acid, and acetic acid.^10,12^ However, these acid–based methods are time-consuming, result in pectin degradation, and cause equipment corrosion.^13^ To address the limitations of traditional extraction methods, emerging technologies and green solvents have been developed for pectin recovery from FSCW. Subcritical water extraction offers high-quality pectin, rapid processing, and reduced acid usage, but its high operational costs have hindered widespread adoption.^14–16^ Enzymatic extraction can minimize the need for feedstock pretreatment, reduce equipment corrosion, and achieve high yields with lower ethanol consumption. Nevertheless, the high price of enzymes hindered the development of this promising technology from laboratory scale to industry.^17–19^

Carbon electrode materials traditionally derived from petroleum-based polymers are difficult to degrade, resulting in environmentally problematic waste.^20^ However, their high cost limits widespread adoption. Consequently, identifying affordable carbon precursors has become a critical research priority. Biopolymer-derived carbon materials have gained considerable attention due to their abundant availability and environmentally friendly properties, making them promising candidates for electrode applications. Biopolymers such as cellulose, lignin, chitin, and starch have been explored as sources of carbon for electrode materials, with these biopolymer-derived carbons exhibiting excellent cycling stability and high-power density.^21^ However, their limited capacity hinders further development.^22^ Thus, there is a need to explore alternative biopolymers or modification techniques to address this limitation.

Yu et al.^23^ reported the pectin conversion into carbon materials through hydrothermal processing, yielding nanospheres and microspheres (10 nm to 3 μm) with a homogeneous structure, demonstrating its potential for use in electrodes. Pectin has also been successfully incorporated into electrode architectures, where its abundant carboxylic and hydroxyl groups provide strong binding forces, helping maintain electrode integrity and preventing the active materials from detaching.^24^

Ionic liquids (ILs), salts that are liquid at low temperature (below 100 °C), present performance advantages in chemical synthesis, catalysis, biocatalysis, electrochemical devices, and as engineering fluids^25^ due to their low vapor pressure, wide temperature range in the liquid form, high stability, ability to dissolve many compounds, and high conductivity. To date, however, no studies have explored the use of ILs for pectin extraction. This study, therefore, pioneers the extraction of pectin from apple pomace using ILs, with the ultimate goal of producing carbon electrodes for battery applications. Quasi-solid-state Na-ion batteries continue to face critical challenges depending on the electrolyte. This includes limited ionic conductivity, high interfacial resistance between electrolytes and electrodes, and mechanical instabilities that facilitate dendrite formation.^26^ Moreover, the development of scalable, low-cost, and sustainable materials remains a key barrier to commercialization. In this context, apple pomace-derived pectin represents a compelling opportunity: its intrinsic functional groups promote Na^+^ transport; its porous architecture enhances interfacial contact and electrolyte uptake; and its biopolymer nature ensures both mechanical flexibility and environmental sustainability. Here, we propose pectin-based membranes as a green and efficient strategy to overcome major bottlenecks in quasi-solid-state Na-ion batteries.

## Materials and Methods

### Feedstock and Chemicals

The dry apple pomace was mechanically ground into 300–500 μm powder (water content 10.39 wt %). Chemicals were purchased from Sigma-Aldrich and used as received, as well as Viscozyme L enzyme. Deionized water (18.2 Ω·cm) was used throughout.

### Synthesis of ILs

ILs were prepared following the same procedure by Nakasu et al.^27^ In brief, the amine [triethylamine, *N*,*N*-dimethylbutylamine (DMBA), and choline (Ch)] was added into a round-bottom flask in an ice bath (0 °C) and continuously stirred with the designated amount of water. A stoichiometric amount of acid (sulfuric acid, acetic acid, or methanesulfonic acid) was added dropwise. The solution was stirred overnight, and the ILs were recovered as a clear and viscous liquid. The water content of the ILs was determined by Karl Fisher titration (Mettler-Toledo V20). The acid-to-base ratio (ABR) was determined by ^1^H NMR (Bruker 400 MHz spectrometer), or, in the case of [HSO_4_] ILs, measured by titration with 0.1 M NaOH with potassium hydrogen phthalate as a primary standard by a G20S Compact Titrator (Mettler-Toledo, Columbus, USA). For the ^1^H NMR analysis, 750 μL of D_2_O and 200 μL of IL were evenly mixed in a small glass vial and then transferred to an NMR tube. Different anions were chosen based on the acidity of the parent acids: sulfuric acid, methanesulfonic acid, acetic acid, and lysine; as for the cations, two protic cations (triethylamine-TEA and DMBA) and one aprotic cation (Ch) were selected in this study. As shown in Figure 1, 10 different ILs, including [Ch]­[Lys], [Ch]­[OAc], [TEA]­[OAc], [DMBA]­[OAc], [Ch]­[MeSO_3_], [TEA]­[MeSO_3_], [DMBA]­[MeSO_3_], [Ch]­[HSO_4_], [TEA]­[HSO_4_], and [DMBA]­[HSO_4_], were used. Figures S1–S7 display the NMR spectra. [DMBA]­[OAc] and [TEA]­[OAc] did not resemble ILs for a few reasons: they presented an acetic-acid-like smell; upon increasing water contents their ^1^H NMR spectra presented variations in some signals shifts due to increasing ionization (data now shown); the same feature was observed with a ionic conductivity, also increasing upon dilution in water (data not shown).

**Figure 1:**
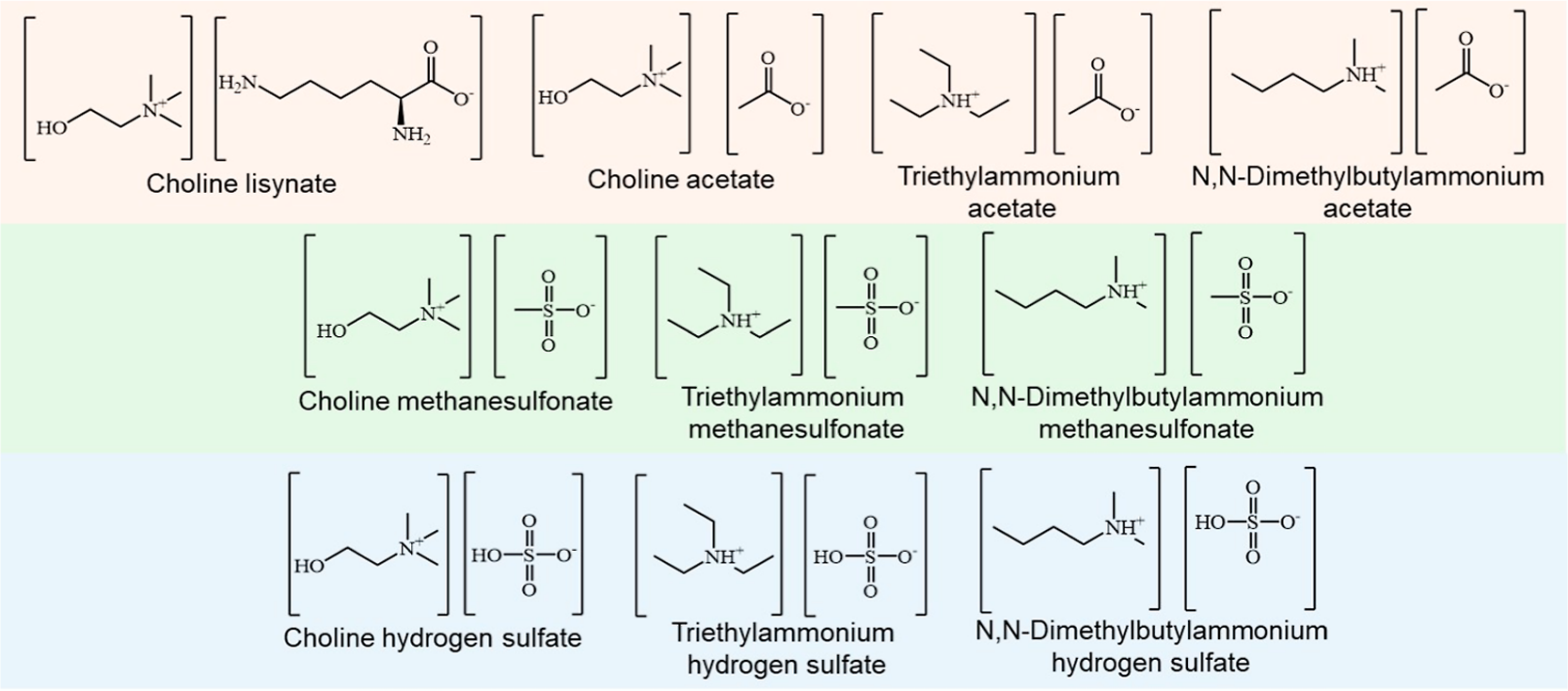
Chemical structure of the synthesized ILs.

### Pectin Extraction Using ILs

Figure 2 shows a flowchart of the pectin extraction process using ILs. The pectin extraction was carried out in a 30 mL glass bottle with a heating plate (Heidolph, model MR Hei-Connect, Schwabach, Germany). For each run, 2 g of apple pomace and 20 g of IL (water content = 30 wt % and ABR = 1.0) were added into the reactor, and then the mixture was heated to the designated temperature with the stirring rate of 400 rpm. The reaction time was recorded after the temperature probe reached the target temperature. When the extraction process finished, the mixture was cooled, and the suspension was centrifuged at 2675 g for 15 min to separate the IL from the solid residue. The solid residue was washed with water (10 mL) twice to remove the residual IL, and the washing solutions were combined. The pectin was precipitated from the IL solution by adding an antisolvent (acetone, ethanol, isopropanol, or butanol) at a ratio of 1:2 (v/v). After the antisolvent was added, the mixture was left to decant further the pectin in a refrigerator at 4 °C for 1 h. Subsequently, the pectin was separated from the solution by centrifugation (g-force at 2675*g*) and was washed twice and then dried overnight at 50 °C in a convection oven (Thermo Scientific Heratherm, model 51028537, Langenselbold, Germany). The pectin yield was determined by eq 1.

**Figure 2:**
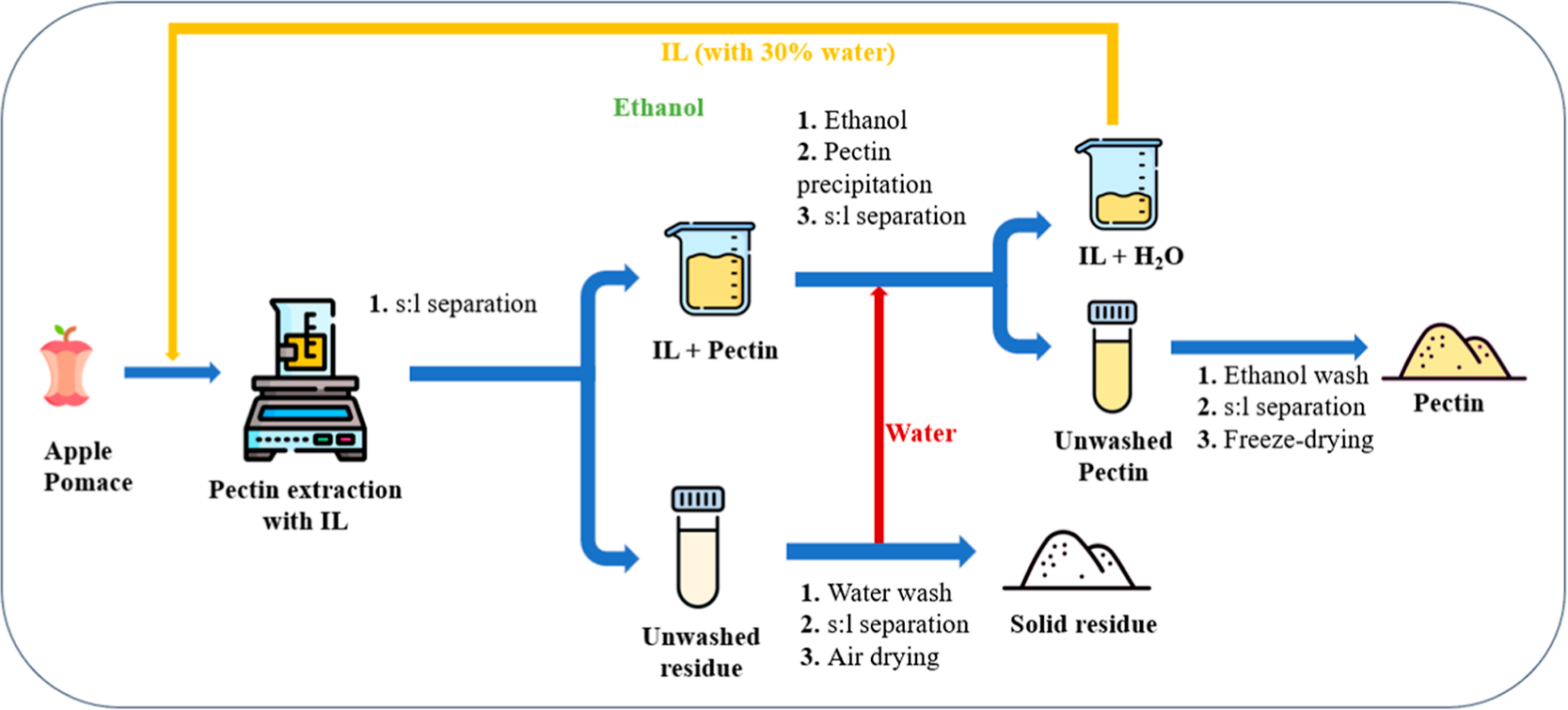
Flowchart of the pectin extraction process.

### Experimental Design

The IL with the highest pectin extraction efficiency was selected for an optimization study, alongside other processing conditions, such as reaction time and antisolvent. A two-level factorial design, resulting in 16 factor-level combinations, determined the effect of parameters such as solids loading, temperature, ABR, and water content on pectin yield, and optimized the extraction to maximize the yield.^28^ The parameters range was based on the conventional extraction parameters^29^ and preliminary tests (data not shown). The ranges of variables were 30–50 wt %, 40–80 °C, 5–15 wt %, and 1–1.1. The experimental design matrix provided 19 extraction runs, including 3 central points. The responses were analyzed on Statistica 10 software by a standard analysis of variance (two-level ANOVA) followed by Fisher’s protected LSD posthoc test at a 0.05 significance level.

### Recycling of IL

The recycling of [DMBA]­[OAc] was performed to investigate the recycling rate, the efficiency along a limited number of cycles, and also the monosaccharide accumulation in the IL. After the pectin was separated from the liquid phase, ethanol and water were removed by rotary evaporation at 50 °C and 50 mbar to recycle the IL, which then had its water content and ABR adjusted (water content of 30 wt % and ABR of 1.1). The recycled IL underwent recycling for four more cycles, totaling five cycles.

### Characterization of Pectin

#### GalA Content

The GalA content of pectin samples was determined by the *m*-hydroxybiphenyl colorimetric method according to Blumenkrantz and Asboe-Hansen^30^ with minor modification by Costa et al.^31^ and the enzymatic hydrolysis method with pectinase. For the *m*-hydroxyphenyl colorimetric method, 200 μL of pectin solution (0.2 g/mL) was mixed evenly with 3 mL of sodium tetraborate solution (0.0125 M in H_2_SO_4_), and the mixed solution was heated at 95 °C for 5 min. After heating, the mixed solution was put into an ice bath to cool down faster. Subsequently, 20 μL of a solution of 0.15% (w/v) *m*-hydroxybiphenyl in 0.5%(w/v) NaOH was added to the former mixture and mixed manually. The absorbance of the obtained solution was measured by UV–vis spectroscopy (Shimadzu, UV-2600, Japan) at a wavenumber of 520 nm. GalA content was determined using a standard curve of GalA monohydrate (valid between 0 and 0.4 mg/mL, *R* ^2^ = 0.99).

For the enzymatic hydrolysis, 50 ± 1 mg of pectin was weighed into a Sterilin tube (30 mL) and 4.9 mL of a mixed solution was added at the same time, consisting of 2.5 mL of 0.1 M sodium citrate buffer (pH = 4.8), 15 μL of cycloheximide antibiotic solution (10 mg/mL in purified water), 20 μL of tetracycline antibiotic solution (10 mg/mL in 70% ethanol), 200 μL of Viscozyme L enzyme, and 2.165 mL of purified water. The Sterilin tube was closed and placed in an incubator at 50 °C for 3 days (250 rpm). After enzymatic digestion, 1 mL of the liquid mixture was sampled through a syringe filter (0.45 μm). All samples were obtained in triplicate with controls (without pectin but 0.1 mL water), and controls were used to determine the residual sugar content. Then, the hydrolysates were analyzed on an HPLC (Shimadzu) equipped with an H-column (5 mM H_2_SO_4_, 0.6 mL/min, and 50 °C). The GalA content was calculated from a standard curve of GalA (valid between 0 and 10 mg/mL, R^2^ = 0.99).

#### Degree of Esterification

The DE and the functional groups of the pectin samples were determined by Fourier-transform infrared (FTIR) spectroscopy (Agilent Technologies, model CARY 630, CA, USA) according to Lin et al.^32^ The FTIR spectra were acquired within the range 600–4000 cm^–1^. The DE was estimated according to the ester carboxyl groups (−COOR) and the free carboxyl groups (−COOH) according to eq 2.

#### Solid-State NMR Spectroscopy

^1^H–^13^C cross-polarization/magic angle spinning (commercial pectin (CP)/MAS) NMR experiments were performed on a Bruker Avance 200 MHz NMR spectrometer, using a Bruker three-channel 4 mm T-3 MAS probe. Radio frequency (rf) field strengths were 60 kHz for ^1^H–^13^C CP, and 80 kHz for ^1^H decoupling. All MAS experiments used a spinning rate of 5 kHz, and CP experiments used a contact time of 1 ms.

#### Thermogravimetric Analysis

The thermogravimetric profiles of the pectin samples were determined with a thermogravimetric analyzer (PerkinElmer, model TGA 8000, US). Before the TGA test, the pectin samples were placed in a 105 °C oven for 1 h to remove residual moisture. Briefly, 3 mg of pectin was weighed into the crucible. The pectin samples were heated from room temperature to 105 °C and maintained for 1 h first, and then heated from 105 to 700 °C at a heating rate of 10 °C/min. Nitrogen atmosphere was guaranteed with a flow rate of 50 mL/min.

### Synthesis of Hard Carbons

Hard carbons were synthesized by hydrothermal carbonization of 4 wt % CP from citrus peel (GalA ≥ 74.0%, Sigma-Aldrich) in 200 mL of deionized water. This solution was placed in a sealed autoclave reactor (50% fill volume) and heated to 230 °C for 6 h under self-generated pressure. The resulting powder was dried under vacuum at 80 °C and then further carbonized at 1300 °C for 2 h under a nitrogen atmosphere.

### Preparation of Porous Membranes

The porous membranes were fabricated via a nonsolvent evaporation method using deionized water as the solvent and ethyl lactate (≥98%, Merck) as the nonsolvent. Pectin extracted from apple pomace and carboxymethylcellulose (CMC, Merck) were blended in 9–10 mL of DI water at weight ratios of 50:50, 60:40, and 70:30 (total polymer mass = 0.10 g). Each mixture was stirred at 50 °C for 7 h, and then 3 mL of ethyl lactate was added, and stirring continued for an additional 3–6 h. The resulting solutions were cast into glass Petri dishes and dried at 46 °C overnight. Once fully evaporated, the membranes were punched into 18 mm disks and vacuum-dried at 60 °C overnight. The 70:30 pectin/CMC formulation was selected for further study due to its superior mechanical stability and optimal porosity.

### Calculations

1 $$Pectin\;yield\;\left(wt\;\%\right)=\frac{dried\;pectin\;mass\;\left(g\right)}{apple\;pomace\;dried\;mass\;\left(g\right)}\times 100$$

2 $$DE\;\left(\%\right)=\frac{peak\;area\;of\;-COOR\;\left(1740\;{cm}^{-1}\right)}{peak\;area\;of\;-COOR\;\left(1740\;{cm}^{-1}\right)+peak\;area\;of\;-COOH\;\left(1616\;{cm}^{-1}\right)}\times 100$$.

## Results and Discussion

### Screening of Extraction Time

The extraction time was evaluated as a variable since a longer processing time requires more energy. Figure S8 shows the pectin yields from different extraction times. Both protic and nonprotic ILs were used for extraction time screening, including [TEA]­[MeSO_3_], [DMBA]­[MeSO_3_], and [Ch]­[OAc]. According to Figure S8, the pectin yield from [TEA]­[MeSO_3_] and [Ch]­[OAc] showed a decrease with the prolonging of extraction time, from 5.6 to 3.8 wt % and from 5.3 to 4.4 wt %, respectively. As time progressed, the neutral sugar side chains of pectin underwent partial hydrolysis reaction in free sugars^33^ or in small molecular weight compounds during the extraction with [TEA]­[MeSO_3_] and [Ch]­[OAc], leading to a reduction in pectin yield. However, [TEA]­[MeSO_3_] and [DMBA]­[MeSO_3_], both protic ILs, showed different relationships between extraction time and yield. There was an increase in the pectin yield for [DMBA]­[MeSO_3_] from 3.6 wt % to 5.0 wt %, probably due to [DMBA]­[MeSO_3_] being more acidic than [TEA]­[MeSO_3_], based on results found by Gschwend et al.,^34^ where, under the same reaction conditions, [DMBA]­[MeSO_3_] extracted more lignin and hemicelluloses than [TEA]­[MeSO_3_]. The use of [DMBA]­[MeSO_3_] favored longer extraction times due to prolonged contact and interaction between the solvent and insoluble pectic substances, enhancing the degree of dissociation and increasing the pectin yield.^35^ In a literature study, the extraction time did not affect the pectin yield while using deep eutectic solvents (DES: Bet-CA) to extract pectin from grapefruit by a centered composite design (CCD)-based response surface methodology.^32^ However, the extraction time was prolonged while the pectin yield decreased in the study of Belan and Israel^36^ and Ma et al.^37^ Longer extraction times may lead to pectin decomposition and lower yield.^38,39^ Therefore, 1 h was selected for further experiment exploration based on the pectin yield.

### Screening of ILs

Ten different ILs were screened as pectin extracting solvents, including different types of anions ([OAc]^−^, [HSO_4_]^−^, [Lys]^−^, and [MeSO_3_]^−^) and cations ([DMBA], [TEA], and Ch). Figure 3 shows the pectin yields from the 10 different ILs. The highest pectin yield achieved up to 9.2 wt % with [DMBA]­[OAc], followed by [Ch]­[Lys] (8.9 wt %) and [Ch]­[OAc] (7.9 wt %), while the lowest was from [TEA]­[OAc] (3.2 wt %). ILs based on [TEA] showed the highest yield, followed by those based on [DMBA], with [Ch]-based ILs yielding the lowest. The highest yields observed within each group were 7.5 and 6.5 wt %, respectively. The effective combination of TEA with strong acids such as H_2_SO_4_ and MeSO_3_H resulted in stronger ionic interactions.

**Figure 3:**
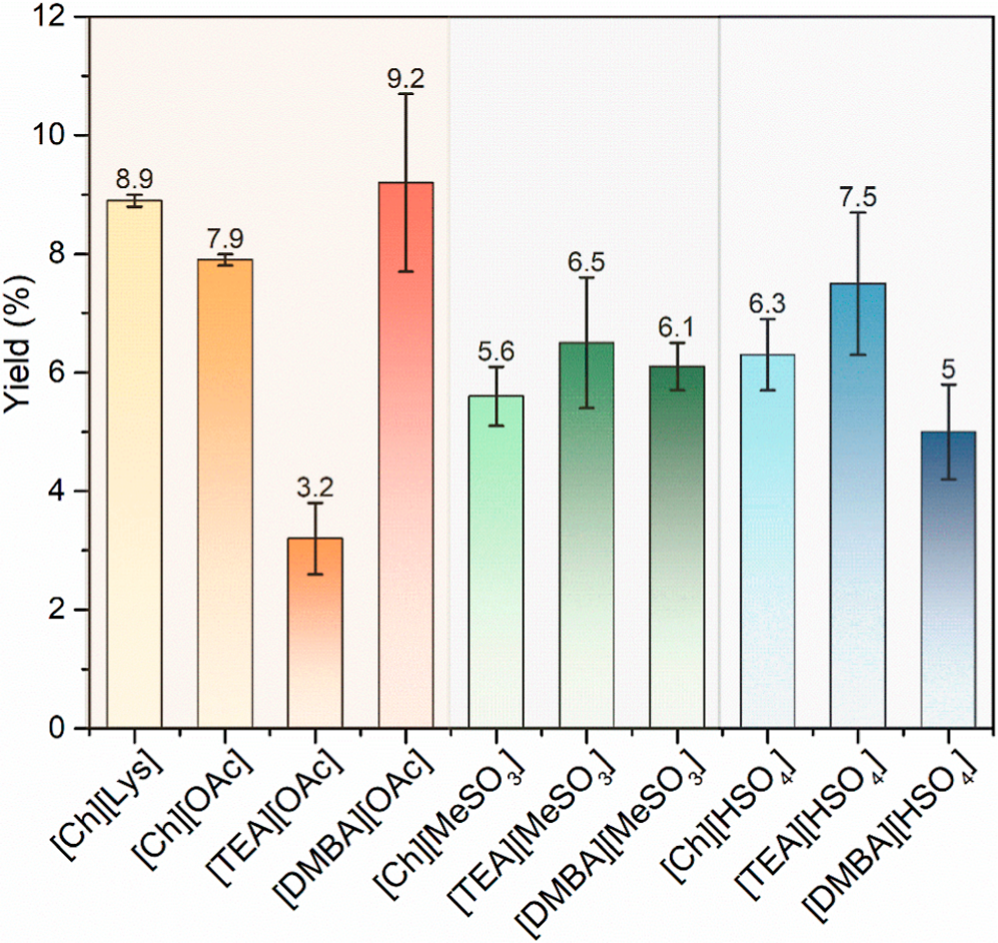
Pectin extraction yield from different ILs (Extraction parameters: ethanol as antisolvents, 10 wt % of solids loading, 30 wt % of water content, 1.0 of ABR, temperature of 60 °C, with the stirring rate of 400 rpm.).

Within the weak anion group ([OAc]^−^), a remarkable difference in pectin yield was observed: [DMBA]­[OAc] achieved the highest yield at 9.2 wt %, whereas [TEA]­[OAc] yielded only 3.2 wt %. This divergent behavior among [OAc]^−^-based ILs may stem from the dynamic ionic equilibrium present in the mixture solution. The combination of [TEA], a weak base, and acetic acid, a weak acid, likely forms a system that is not a fully dissociated IL, but rather a complex mixture containing oligomeric ions,^40–43^ with distinct physicochemical properties. A similar mechanism may apply to [DMBA]­[OAc]. However, compared to [TEA]­[OAc], [DMBA]­[OAc] possesses a more asymmetric, bulky, and hydrophobic cationic structure. The size and hydrophobicity of the ammonium group play critical roles in its interaction with pectin during extraction; the bulkier butyl substituent in [DMBA]­[OAc] likely enhances interactions with hydrophobic domains of pectin, thereby facilitating its release from the biomass matrix. Gschwend et al.^34^ reported that [DMBA]­[HSO_4_] outperformed [TEA]­[HSO_4_] in the pretreatment of pine, likely due to the asymmetric structure of DMBA, presenting a more accessible proton than the bulkier TEA, ultimately increasing the likelihood of hydrogen bond interactions with biomass. Yao et al.^44^ also reported a better performance of [DMBA]­[ HSO_4_] over [TEA]­[HSO_4_] on the removal of heavy metals from sewage sludge, likely due to the reduced steric hindrance facilitating proton accessibility.

Overall, the pectin extraction yield was governed by the combined physicochemical properties of the IL cation and anion. Although the extraction efficiency of pectin from [DMBA]­[OAc] was comparable to that from [Ch]­[Lys] and the standard deviation from [DMBA]­[OAc] was higher, the latter was chosen for the optimization experiments for two reasons. First, Ch lysinate synthesis, despite being a protic IL and therefore easier than aprotic IL synthesis (such as for imidazolium-based ILs), is still problematic in terms of water consumption, and the reaction between [Ch]­[OH] and lysine is slow due to the low acidity of the amino acid. Second, the starting materials costs and environmental footprint impact the final IL synthesis cost and impact. Lysine and Ch hydroxide are more expensive and incur a greater production impact than DMBA and acetic acid.

### The Screening of Antisolvents

On an industrial level, alcohol precipitation is a commonly employed method to recover pectin from solutions, as it is well-suited for large-scale production of this polysaccharide.^33^ In this section, alternative precipitation agents were evaluated to enhance the pectin recovery. Five protic solvents were tested. In decreasing degrees of polarity, ethanol, isopropanol, 1-butanol, pentanol, hexanol, and one aprotic solvent, acetone, were also tested. The extraction and precipitation parameters were consistent with those used during IL screening, except that the antisolvent concentration was varied for each run. Table 1 displays the yield and visual characteristics of the pectin products obtained with each antisolvent. Notably, pentanol and hexanol failed to induce pectin precipitation due to their low polarity and immiscibility with water.

**Table 1:**
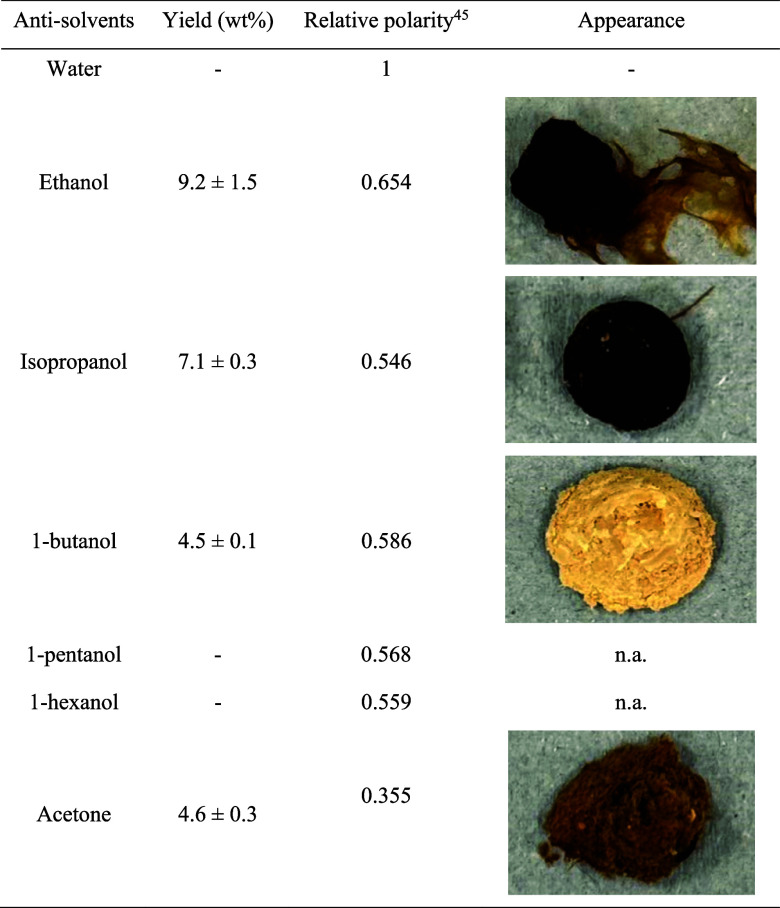
Yield and Appearance of Pectin Products from Different Antisolvents^a^ ^45^

a Processing condition: [DMBA]­[OAc] (water content = 30 wt %, ABR = 1.01), 10 wt % of solids loading, 60 °C heating for 1 h and stirring at 400 rpm.

As shown in Table 1, ethanol yielded the highest pectin recovery (9.2 wt %). Among the tested n-alcohols, a decrease in the pectin yield correlated with decreasing polarity of the precipitation agent. de la Hoz Vega et al.^46^ reported a similar result regarding the relationship between solvent polarity and pectin yield, where methanol, ethanol, and 1-propanol were used for pectin precipitation. Pectin precipitated with 1-butanol exhibited a notably lighter color compared to those obtained with other antisolvents. This outcome can be attributed, in part, to the gelling behavior of ethanol; as reported by Happi Emaga et al.,^47^ ethanol can coprecipitate nonpectin compounds. On the other hand, the polarity of the antisolvent also influenced the precipitation of dark-colored compounds in pectin using ethanol. Common pectin components such as polyphenols and pigments present lower polarity and therefore are soluble in 1-butanol but not in ethanol, isopropanol, or acetone; once extracted, these compounds did not darken the pectin upon drying in the oven.

FTIR analysis was employed to investigate the chemical bonding characteristics of extracted pectin, as shown in Figure 4a. Despite the darker visual appearance of the ethanol-precipitated pectin, its spectral features closely resembled those of CP, indicating comparable structural integrity. Consequently, pectin yield was prioritized over appearance, and ethanol was selected as the antisolvent for further optimization using [DMBA]­[OAc]. To compare the local carbon environments in optimized IL-extracted pectin and CP, solid-state ^1^H–^13^C CP/MAS NMR spectroscopy was performed (Figure 4c). Both samples displayed characteristic carbohydrate pectic resonances between 50 and 100 ppm, attributable to hydroxylated carbons, as well as a prominent signal at 170 ppm, corresponding to carbonyl groups associated with unsaturated functionalities.^48,49^ In the IL-extracted pectin spectrum, two additional peaks were observed at 35 and 40 ppm, which are assigned to acetylated hydroxyl carbons and methylated carboxylate groups, respectively.^50^ For clarity, the assignments of the numbered resonances in Figure 4c are as follows: signals between 50 and 100 ppm correspond mainly to C2, C3, and C5 carbons in GalA units; the resonance at ∼170 ppm arises from carboxyl carbons (C6) of the galacturonic backbone; the peak near 35 ppm is assigned to acetyl substituents attached to hydroxyl groups, while the signal at ∼40 ppm corresponds to methyl groups esterifying carboxyl functions. These assignments are consistent with previous reports on the pectin structure obtained by solid-state NMR. The absence of extraneous resonances, coupled with the distinct presence of these functional-group signals, underscores the high chemical purity of the IL-extracted pectin.

**Figure 4:**
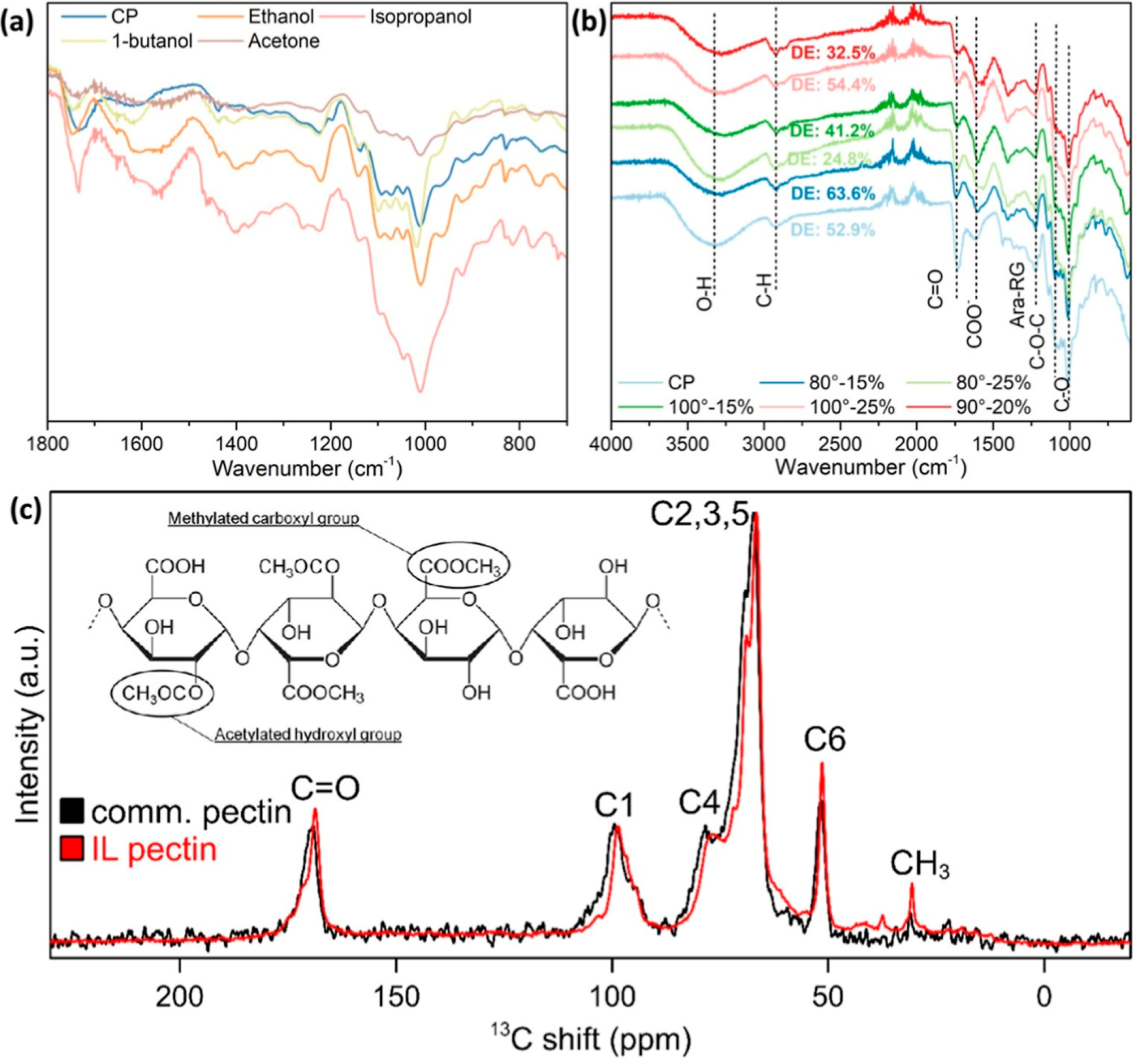
(a) The FTIR spectra of pectin products from different antisolvents; (b) FTIR spectra of pectin samples under different extraction parameters; (c) ^1^H–^13^C CP/MAS NMR spectrum of optimized pectin isolated by [DMBA]­[OAc] and ethanol compared to CP extracted from citrus peel.

### Conditions Optimization with [DMBA]­[OAc]

Table 2 shows the pectin yield using [DMBA]­[OAc] as a function of the design of experiments variables. Equation 3 provides the pectin yield at a 95% confidence level and a regression coefficient (*R* ^2^) of 98.32%. The model considered the highest adjusted *R* ^2^, being 96.65%. The ANOVA data shown in Table S1 were assessed by Fisher’s test. The model was significant and predictive in the designed range of parameters. The lower *p*-value (*p* < 0.05) proved the higher statistical significance of individual parameters.^51^

3 Pectinyield(wt%)=5.54+3.80×T+1.68×SL+0.28×ABR−0.23×WC×SL+1.33×T×SL+0.28×T×ABR−0.20×SL×ABR−0.45×WC×SL×ABR

where *T* is the temperature; SL is solid loading; WC is the water content; and ABR is the acid-to-base ratio.

**Table 2:** Two-Level Factorial Design of Pectin Extraction

	variables				response
exp.	water content (wt %)	temperature (°C)	solid loading (wt %)	ABR	yield (wt %)
1	50	80	5	1.1	7.0
2	50	80	15	1.1	8.5
3	30	40	5	1	3.7
4	50	40	15	1.1	3.7
5	30	80	15	1.1	9.6
6	30	80	5	1.1	6.2
7	30	40	15	1.1	4.3
8	30	40	15	1	3.7
9	40	60	10	1.05	5.1
10	40	60	10	1.05	4.9
11	50	80	15	1	9.1
12	30	80	15	1	9.0
13	40	60	10	1.05	5.0
14	50	80	5	1	5.3
15	50	40	15	1	4.0
16	30	80	5	1	5.7
17	50	40	5	1	3.6
18	30	40	5	1.1	3.4
19	50	40	5	1.1	3.6

The Pareto chart in Figure 5a shows the effect of the parameters on the response. The temperature had the strongest influence on pectin yield, followed by solids loading. Huo et al.^52^ and Lin et al.^32^ reported similar results, where high temperatures were crucial for breaking the pectin bonds with the cell wall, thereby increasing its solubility and yield. Acidic solutions were used for pectin extraction in conventional approaches, for example, Spinei and Oroian^53^ reported that a lower pH of the extraction solvent led to higher pectin yield. Therefore, the ABR and water content of ILs were adjusted during the synthesis procedure, which may have affected the acidity of ILs. Water content and ABR of [DMBA]­[OAc] exhibited no significant influence on the pectin yield. The distinctive and strong solubility of ILs overwhelmed the effect of acidity on the pectin yield.

**Figure 5:**
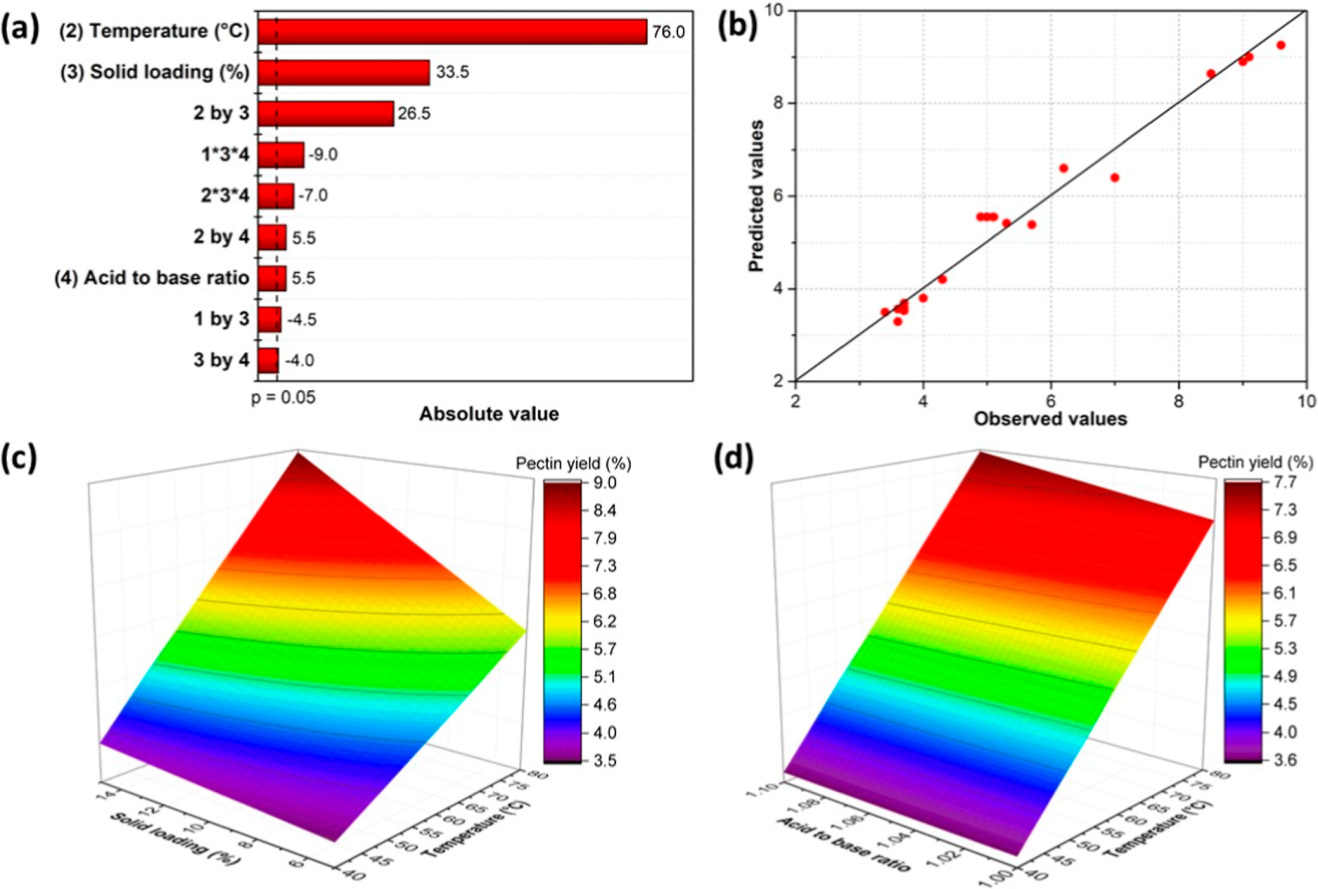
(a) Pareto chart: (1) temperature, (2) solids loading, (3) water content, (4) ABR; (b) observed versus expected values; (c) response surface—solids loading versus temperature; (d) response surface—ABR versus temperature.

Figure 5b displays the predicted data versus the experimental data, suggesting a good fit of the model. The influence of extraction temperature on pectin yield was more pronounced at higher temperatures (Figure 5c), while there was no apparent difference from a low acid-to-base ratio to a high acid-to-base ratio (Figure 5d). An increase in solid loading enhanced pectin yield at higher temperatures. However, Costa and Forster-Carneiro^54^ reported that high pectin yield could be obtained with low solids loading, while for Huo et al.,^52^ the extraction efficiency increased with the increase of solids loading, and then decreased after a certain point. Reported effects of solid loading vary across studies, likely due to differences in raw materials and extraction solvents.

Based on the results, the extraction temperature and solids loading were investigated up to 100 °C and 25 wt %, respectively. Table 3 displays the yield and GalA content of the additional samples. Pectin yields obtained at 100 °C were consistently lower than those at 80 °C across all solid loading ratios, likely due to thermal degradation of the pectin chains. Pereira et al.^55^ demonstrated that the molecular weight of pectin extracted using subcritical water and pressurized natural deep eutectic solvents decreased with increasing extraction temperatures. In this study, a solid loading of 25 wt % resulted in higher pectin yields compared to 15 wt %. However, despite the improved yield at higher solid loadings, the resulting pectin may exhibit a reduced purity.

**Table 3:** Yield and Characteristics of Pectin from the Second Time of Two-Level Factorial Design

temperature/solids loading	100 °C-15%	100 °C-25%	80 °C-15%	80 °C-25%	90 °C-20%	commercial pectin
pectin yield (wt %)	5.3 ± 0.8	7.4 ± 0.8	8.0 ± 0.5	8.6 ± 0.1	8.1 ± 0.6	
GalA content (wt %)^a^	81.7 ± 0.1	61.6 ± 1.0	73.9 ± 0.7	65.6 ± 1.0	72.2 ± 0.0	83.3 ± 0.1
GalA content (wt %)^b^	62.6 ± 1.0	56.4 ± 0.7	65.9 ± 0.4	56.4 ± 0.7	52.1 ± 0.1	98.9 ± 0.4

a GalA content obtained from *m*-hydroxyphenyl colorimetric method.

b GalA content obtained from enzyme digestion method.

For the enzyme digestion and *m*-hydroxyphenyl colorimetric methods, the GalA content from low solid loading reactions was higher than that from high solid loading reactions, supporting the hypothesis that higher pectin yields are associated with lower purity. A reduced GalA content indicates a greater presence of nonpectic components, such as proteins, starch, or sugars.^56^ Notably, the GalA content determined by the two methods differed significantly. Colorimetric methods depend on the complete hydrolysis of pectic substances into GalA, along with hexoses and pentoses, under concentrated acid conditions. Uronic acids react with sulfuric acid to form 5-formyl-2-furancarboxylic acid, while hexoses and pentoses yield 5-hydroxymethyl-2-furancarboxaldehyde and 2-furancarboxaldehyde, respectively. These intermediates subsequently react with *m*-hydroxyphenyl to generate colored complexes, typically ranging from red to pink.^57^

Specifically, the reaction involving d-GalA exhibits maximum absorbance within the 520–530 nm wavelength range.^58^ Consequently, the presence of unknown chemical derivatives may influence UV absorbance, potentially leading to an overestimation of GalA content compared with the enzymatic digestion method. In addition to the CP sample, the highest GalA content determined by the *m*-hydroxyphenyl colorimetric method was found in the pectin extracted at 100 °C with 15% solid loading, whereas the enzymatic method indicated the highest content for the sample obtained at 80 °C with 15% solid loading, highlighting differences arising from the underlying reaction mechanisms. The CP samples exhibited GalA content of almost 98.9 wt %, contrary to the 65–75 wt % reported in previous studies.^7,59^ The optimized pectin extraction occurred under the following conditions: temperature—80 °C, solids loading, 15 wt %; ABR—1.1 using [DMBA]­[OAc], water content—30 wt %.

### Pectin Characterization: Functional Groups and Thermal Stability

Figure 4b shows the FTIR spectra highlighting the functional groups and molecular structures of the pectin samples. The absorption band at 3320 cm^–1^ represented the stretching vibration of O–H group.^14^ The bands at 2930–2920 cm^–1^ were assigned to the C–H stretching vibration of –CH_2_– and –CH_3_ groups.^29^ The absorption bands between 1800 and 1500 cm^–1^ corresponded to the absorption of carboxylic acid and ester groups found in pectin molecules.^55^

The vibrations of the C–O stretching of the methyl esterified carboxylic groups and the asymmetric vibrations of stretching of the carboxylate group of pectin molecules were located at 1740 and 1616 cm^–1^, respectively. The DE of the pectin samples was estimated based on the area ratio of these two peaks, as reported in Figure 4b. The DE of pectin samples extracted using [DMBA]­[OAc] varied between 24.8 and 63.6%. The DE in general decreased with an increase in temperature. While DE generally decreased with increasing temperature, at 80 °C, a higher solid loading reduced DE, whereas at 100 °C, the opposite trend was observed.

The DE varied depending on the extraction solvent. It ranged from 24.8% at 80 °C and 25 wt % solid loading to a maximum of 63.6% at 80 °C and 15 wt % solids loading. The difference in DE occurred due to the extraction condition, resulting in a different de-esterification effect. The band at 1216 cm^–1^ was assigned to C–C stretching vibrations in the ring structure of the pectic polysaccharide,^32^ while the band around 1100 cm^–1^ suggested the presence of furanose structures as well as α- and β-pyranose rings.^54^ The band near 1000 cm^–1^ was attributed to the skeletal C–O vibration bands of the glycosidic linkage, a characteristic feature of the pectin backbone.^52^

Figure 6a displays the thermal stability of the pectin samples. The thermal degradation of pectin obtained from [DMBA]­[OAc] occurred in three phases: 105–180 °C, 180–400 °C, and 400–700 °C. During the first phase, the mass loss is attributed to the evaporation of water and other volatile substances. However, the weight loss of the first stage was low due to the 105 °C oven drying before testing. The second phase demonstrated significant mass loss, attributed to the pyrolysis of heteropolysaccharide chains, including decarboxylation and cleavage of functional groups.^60^ Furthermore, there could be more volatile compounds in pectin samples extracted by [DMBA]­[OAc], indicating the lower purity. The CP started its degradation process at a higher temperature than the pectin samples extracted using ILs, indicating that there are more volatile components in the IL-extracted pectin, such as DMBA itself.

**Figure 6:**
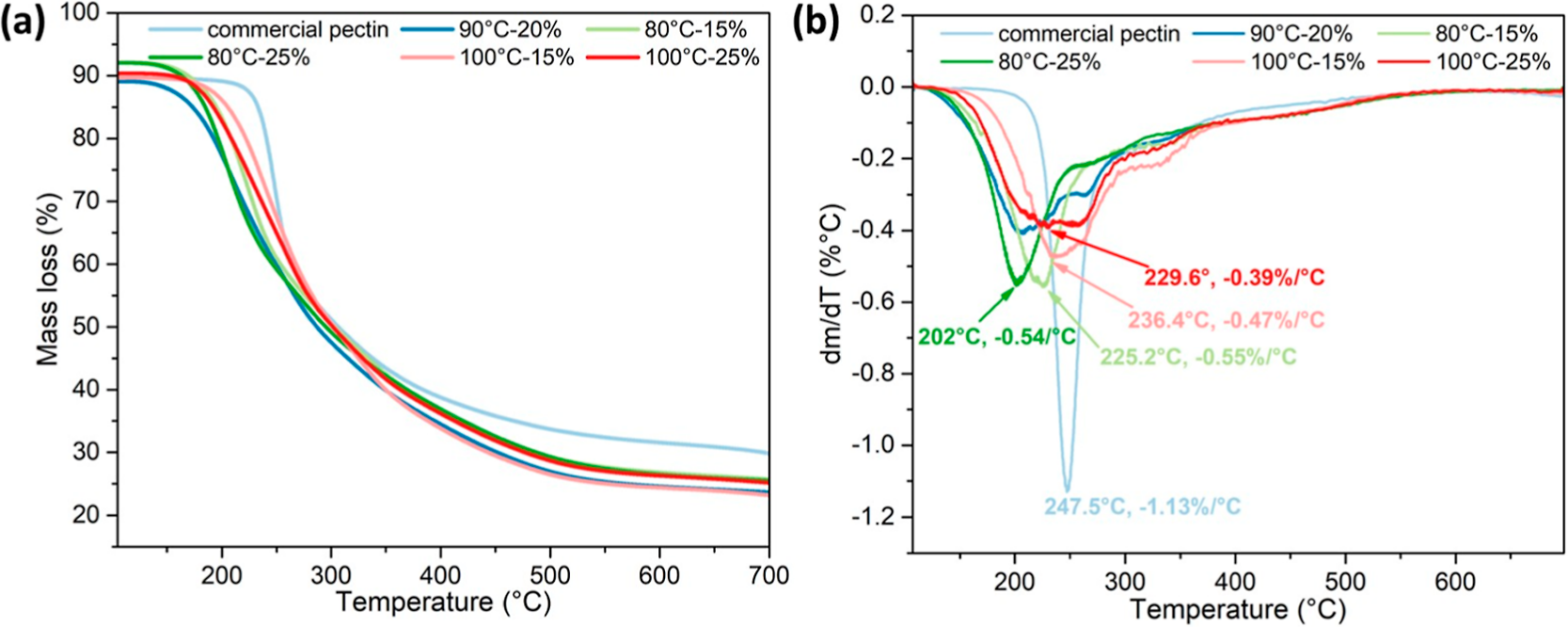
(a) TGA curves of pectin samples; (b) DTG curves of pectin samples.

The third degradation phase began at 400 °C, where the pectin samples underwent carbonaceous residue (char) degradation, resulting in a relatively lower mass loss during this stage. At 650 °C, the CP exhibited a weight loss of about 70 wt %, whereas the pectin samples extracted using [DMBA]­[OAc] showed a weight loss of approximately 85 wt %, consistent with the values reported by Santos et al.^60^ Figure 6b details the derivative thermogravimetric (DTG) curves of the different pectin samples. A narrower DTG peak generally indicates the thermal decomposition of a more homogeneous matrix,^61^ like CP that exhibited a sharp peak at 247.5 °C. In contrast, all IL-extracted pectin samples displayed broader peaks below 240 °C, indicating a less uniform composition. The CP began to degrade later than pectin samples extracted by [DMBA]­[OAc]. Einhorn-Stoll et al.^61^ explained that pectin with lower DE values has more free carboxyl groups available to form additional hydrogen bonds, which can accelerate the degradation of pectin chains. Considering the maximum degradation rate of pectin products extracted by IL, a higher extraction temperature and a higher solids loading led to a lower maximum degradation rate due to the lower amount of residual volatile compounds. On the other hand, a higher extraction temperature and a lower solids loading led to a higher temperature corresponding to the maximum degradation rate.

### Recycling of [DMBA]­[OAc]

Recycling of [DMBA]­[OAc] was carried out five times. Table 4 shows the pectin yield, residue yield, and IL recovery rate. The pectin yield gradually declined from 11.1 to 8.6 wt %, while the solid residue yield increased from 22.8 to 32.8 wt % by the fifth cycle, indicating a reduction in extraction efficiency. Notably, a marked increase in pectin yield was observed during the second extraction cycle (18.9 wt %), deviating from the expected declining trend over successive recycles. This unexpected result may be attributed to residual pectin remaining in the biomass after the initial extraction or to physicochemical alterations in the IL following its first use, which may have transiently enhanced its extraction efficiency.^62^ Alternatively, minor fluctuations in the experimental conditions or analytical variability cannot be ruled out. HPLC analysis confirmed the accumulation of monosaccharides, such as sucrose, glucose, and fructose, with their concentrations increasing progressively across recycling cycles, as reported in Table S2. While the accumulation of sugars and other compounds in the recycled IL likely impaired extraction efficiency, their subsequent recovery could add value to the recycling process by separating different sugars. For [DMBA]­[OAc], its more molecular nature allows for sugar recovery via evaporation, presenting an opportunity for additional valorization of the recycling process.

**Table 4:** Five Runs of the [DMBA]­[OAc] Recovery Process

cycle	1	2	3	4	5
pectin yield (wt %)	11.1 ± 0.4	18.9 ± 0.3	10.8 ± 0.8	9.8 ± 0.7	8.6 ± 0.5
solid residue yield (wt %)	22.8 ± 0.9	21.7 ± 0.3	29.8 ± 0.5	21.5 ± 0.4	32.8 ± 0.7
IL recovery rate (wt %)	92.9 ± 1.1	91.3 ± 1.8	86.2 ± 4.4	88.3 ± 0.3	82.6 ± 2.9

The recovery rate of IL decreased from 92.9 to 82.6 wt %, indicating that part of IL remained in the product, byproducts, or the waste liquid. Elemental analysis and ^1^H NMR were performed to trace the lost IL, as shown in Table S3 and Figure S9. ABR were determined based on the NMR spectra. There was an increase in ABR from 1.1 to 1.3 for each run, indicating that [DMBA]­[OAc] was volatilized and part of the DMBA was released during the recovery process. However, the N content of the waste liquid from evaporation was less than 0.1 wt %. The bulk of water and ethanol in the waste may have led to a lower proportion of nitrogen. On the other hand, nitrogen was found in the pectin and solid residue, corroborating the findings of Wahlström et al.^63^ that 2.9 wt % of [Ch]Cl was found to remain on the product. The washing procedure or purification of the product should be improved to remove the adhered IL for further application, such as dialysis,^32,52^ ionic exchange, and nitration, as well as combined methods.^64^ Although the pectin products extracted from IL are not nitrogen-based, this polysaccharide can be used as a precursor for carbon-based electrode materials. According to Gao et al.,^65^ carbon materials doped with nitrogen synthesized through the simultaneous carbonization and activation of polypyrrole-coated paper towels, exhibited exceptional performance as an electrode in a supercapacitor, displaying a high specific capacitance in an alkaline medium. Furthermore, the ^1^H NMR spectra of [DMBA]­[OAc] showed that no major byproducts were produced during the extraction process.

### Application of Ionic-Liquid-Extracted Pectin Membranes in Quasi-Solid-State Na-Ion Batteries

The prepared membrane (presoaked for several hours in 1 M NaPF_6_ dissolved in EC/DMC) was electrochemically tested by ionic conductivity, electrochemical stability window (ESW), and Na-ion transference number (*t*_Na^+^_) measurements. The ionic conductivity was measured by recording EIS spectra on the cell SS|soaked membrane|SS, while the ESW was measured by performing linear sweep voltammetry (LSV) on the cell SS|soaked membrane|Na, where SS is stainless steel and Na is metallic sodium.^66,67^ The ionic conductivity was found to be ∼1.3 × 10^–3^ S cm^–1^ (recorded EIS spectra are presented in Supporting Information, Figure S10), and the ESW was found to be ∼5.4 V (Figure 7a). The *t*_Na^+^_ was measured on a cell setup Na|soaked membrane|Na with a potentiostatic polarization (Δ*V*) of 7 mV, and the resulting steady-state currents (*I*_0_ and *I*_ss_) were recorded.^68^ The *t*_Na^+^_ was evaluated from the equation

4 $$t_{{Na}^{+}}=\frac{I_{ss}\left(\Delta V-R_{0}I_{0}\right)}{I_{0}\left(\Delta V-R_{ss}I_{ss}\right)}$$

where the interfacial resistances before and after DC polarization (*R*_0_ and *R*_ss_, respectively) were obtained using AC impedance spectroscopy measurements.^69^ The obtained chronoamperometry curve is shown in Figure 7b, and the measured *t*_Na^+^_ was found to be ∼0.74. The measured impedance spectra before and after polarization are represented in Figure S11.

**Figure 7:**
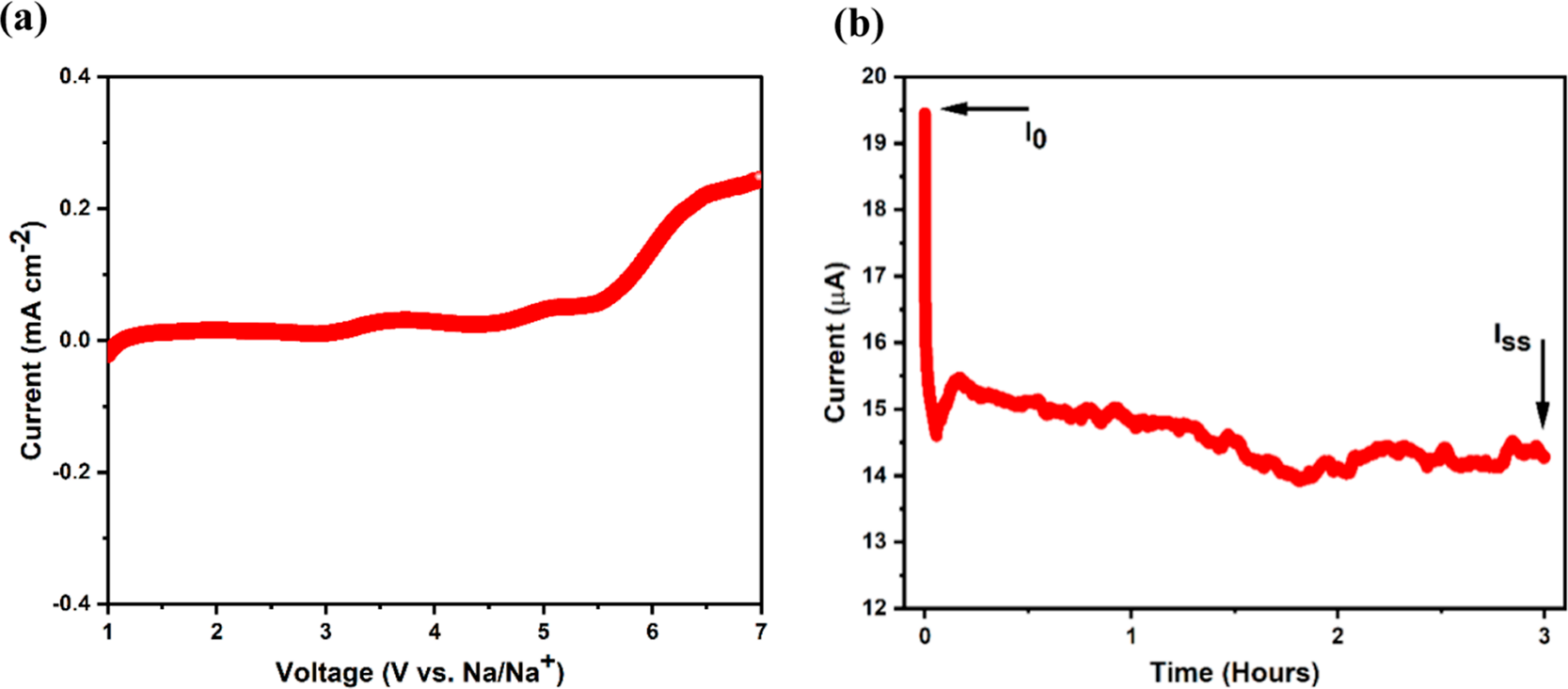
(a) LSV curve for the evaluation of ESW. (b) Obtained chronoamperometry curve for the evaluation of *t*_Na^+^_.

Figure 8a shows the cast membrane and its SEM micrograph, revealing an interconnected porous network that enhances liquid-electrolyte wetting and Na^+^-ion transport. This architecture supports electrolyte retention and continuous ion conduction.^70^ To evaluate battery performance, we assembled half-cells using a hard-carbon anode derived from CP.^71^ The galvanostatic charge–discharge profile at C/20 (Figure 8b) exhibits the typical hard-carbon behavior: a gradual slope and a plateau below ∼0.1 V vs Na/Na^+^.^72^ The first discharge capacity reached ∼129 mAh g^–1^, while the charge capacity was ∼76 mAh g^–1^, yielding an initial Coulombic efficiency (ICE) of ∼60%, a solid result for a sustainable quasi-solid-state cell. The higher first-discharge capacity compared to the charge capacity is attributed to the formation of the solid–electrolyte interphase (SEI) and to irreversible Na^+^ insertion into defect sites of the hard carbon anode, both common phenomena in sodium-ion batteries. These processes typically lead to a reduced ICE that stabilizes in subsequent cycles, as also observed in this study.

**Figure 8:**
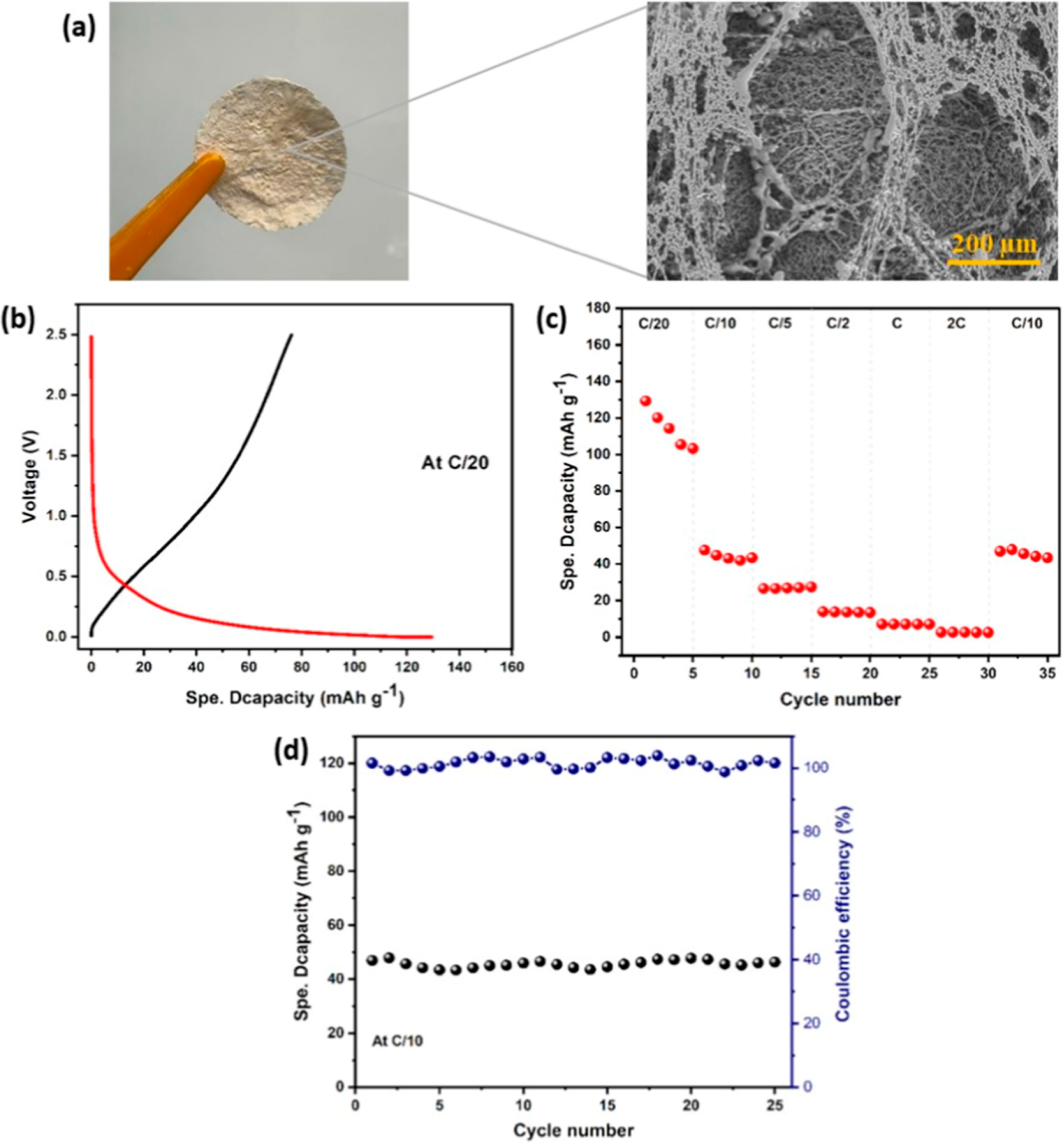
(a) Photograph of the porous pectin/CMC membrane and corresponding SEM micrograph (×250 magnification). (b) Galvanostatic charge–discharge profiles at C/20. (c) Rate capability at various C-rates (C/20 to 2 C). (d) Cycling performance (capacity retention) and Coulombic efficiency over 25 cycles at C/10.

Rate capability tests from C/20 to 2 C (Figure 8c) show the expected capacity drop at higher currents due to transport limitations and polarization, yet the membrane remains intact. Upon returning to C/10, the capacity fully recovers, confirming structural stability and reversibility. Cycling over 25 cycles at C/10 (Figure 8d) demonstrated excellent capacity retention and a stable Coulombic efficiency (∼99–100%), indicative of a robust SEI. Recent studies underscore the growing potential of biomass-derived components in sodium-ion batteries. Matei Ghimbeu et al.^73^ showed that purified lignin can yield hard carbons with stable cycling and capacities up to 284 mAh g^–1^. Mushtaq et al.^74^ demonstrated that optimizing the lignin–PLA ratio in electrospun nanofibers produces conductive 3D networks with long-term stability (170 mAh g^–1^ after 900 cycles). Complementarily, Conder et al.^75^ reported that chitin- and chitosan-derived hard carbons deliver ∼280 mAh g^–1^ at C/10, with performance shaped by porosity and impurities; acid treatment further enhanced the stability of chitosan carbons. Together, these advances highlight the versatility of renewable biopolymers and support the promise of pectin-derived porous membranes for next-generation Na-ion batteries.

## Conclusion

ILs were successfully used to extract pectin from apple pomace. In addition, the extracted polysaccharide was evaluated as a carbon electrode for energy storage systems. The low-cost protic solvent [DMBA]­[OAc] stood out among the 10 ILs evaluated, providing a pectin yield of 9.6 wt % under the optimized condition. High extraction temperatures and solid loadings led to the degradation of pectin and the presence of impurities, respectively. The fabricated quasi-solid-state Na-ion half-cell exhibited excellent cycling stability with Coulombic efficiency close to ∼99–100% for 25 cycles. Thus, research using ILs as a pectin-extracting solvent has significantly expanded the valorization of waste from the food processing industry. The use of low-cost feedstock, environmentally friendly solvents, and promising applications offers great potential for the development of biorefineries. Our findings demonstrated that apple pomace-derived pectin can effectively serve as a renewable alternative to petroleum-based polymers for quasi-solid-state Na-ion battery membranes. This strategy contributes to the sustainability of energy storage materials and also helps overcome two key challenges of solid-state electrolytes: enhancing ion transport through porous, hydrophilic architectures and reducing the environmental burden associated with synthetic polymers. While further optimization and electrochemical benchmarking are required, this proof-of-concept highlights how waste-derived biopolymers can address both performance and sustainability gaps in next-generation Na-ion batteries.

## Supplementary Material



## Notes

^1^H NMR spectra of different ILs used for pectin extraction and the recycled IL; the pectin yield from different extraction conditions; the ANOVA results for a quadratic model of pectin yield; the concentration of different monosaccharides in the recycled IL; and the element analysis of different parts during the pectin extraction (PDF)

## Nomenclature

IL(s): ionic liquid(s)
[Ch]­[HSO_4_]: choline hydrogen sulfate
GalA: galacturonic acid
[Ch]­[MeSO_3_]: choline methanesulfonate
DE: degree of esterification
[DMBA]­[HSO_4_]: *N*,*N*-dimethylbutylammonium hydrogen sulfate
ANOVA: analysis of variance
[DMBA]­[OAc]: *N*,*N*-dimethylbutylammonium acetate
*R* ^2^: coefficient of determination
[DMBA]­[MeSO_3_]: *N*,*N*-Dimethylbutylammonium methanesulfonate
[Ch]­[Lys]: choline lysinate
[TEA]­[HSO_4_]: triethylammonium hydrogen sulfate
[Ch]­[OAc]: choline acetate
[TEA]­[MeSO_3_]: triethylammonium methanesulfonate
ABR: acid-to-base ratio
[TEA]­[OAc]: triethylammonium acetate
