# Supplementary material for: Pectin Extracted by a Recyclable Molecular Mixture: A Promising Material for Porous Membranes in Quasi-Solid-State Na-Ion Batteries
Source: ACS Sustain Chem Eng. 2025 Oct 10;13(42):18061–74. doi: 10.1021/acssuschemeng.5c07306 (PMC12570262; doi:10.1021/acssuschemeng.5c07306)
Supplement: Supplementary file 1 [file sc5c07306_si_001.pdf]

Supporting Information

Pectin extracted by a recyclable molecular mixture: a promising material for porous membranes in quasi-solid-state Na-ion batteries

Wenli Wang[1,2], Pedro Y. S. Nakasu[2*], Josiel Martins Costa[2], Francesco D'Acierno[3], Niyaz Ahmad[4], Maria Magdalena Titirici[2], Daniele Pontiroli[4], Mauro Riccò[4], Changwei Hu[1*], Jason P. Hallett[2]

[1] Key Laboratory of Green Chemistry and Technology, Ministry of Education, College of Chemistry, Sichuan University, 29 Wangjiang Road, Chengdu, Sichuan 610064, PR China

[2] Department of Chemical Engineering, Imperial College London, London, SW7 2AZ, UK

[3] Department of Chemical Engineering, KU Leuven, Gebroeders de Smetstraat 1, 9000 Ghent, Belgium

[4] Nanocarbon Laboratory, Department of Mathematical, Physical and Computer Sciences, University of Parma, Parco Area delle Scienze 7/A, 43124, Parma, Italy

* Corresponding author: p.souza-nakasu17@imperial.ac.uk, changweihu@scu.edu.cn

Number of Pages: 9   Number of Figures: 11   Number of Tables: 3

Content

Supporting Information..1
Figure S1. The ^{1}H spectra of [DMBA][OAc] 3
Figure S2.The ^{1}H spectra of [DMBA][$MeSO_3$] 3
Figure S3. The ^{1}H spectra of [Ch][$MeSO_3$] 4
Figure S4. The ^{1}H spectra of [Ch][OAc]...4
Figure S5. The ^{1}H spectra of [Ch][Lys] 5
Figure S6. The ^{1}H spectra of [TEA][OAc] 5
Figure S7. The ^{1}H spectra of [TEA][$MeSO_3$] 6
Figure S8. The yield of Pectin from different extraction time. 6
Figure S9. The ^{1}H NMR spectra of recycled [DMBA][OAc]......................................7
Figure S10. Recorded EIS spectra for ionic conductivity measurement.......................7
Figure S11. Recorded EIS spectra before and after DC polarization............................8
Table S1. ANOVA results for a quadratic model of pectin yield 8
Table S2. The concentration of different monosaccharide in the recycled IL (mg/mL) 8
Table S3. The element analysis of different part during the pectin extraction process.9

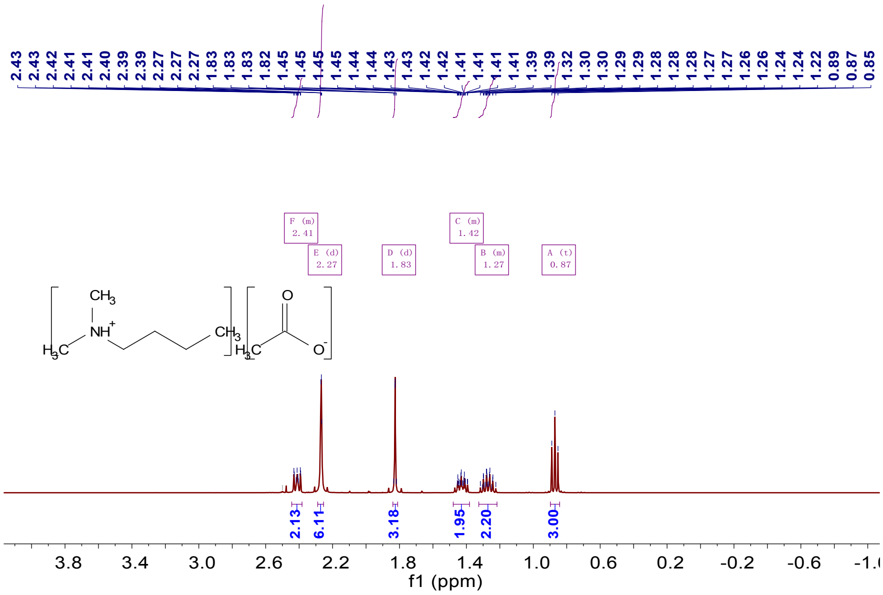

Figure S1. The ^{1}H spectra of [DMBA][OAc]

[DMBA][OAc] (400 MHz, DMSO-d_6): δ 2.44 – 2.38 (m, 2H), 2.27 (d, J = 1.2 Hz, 6H), 1.83 (d, J = 0.7 Hz, 3H), 1.48 – 1.38 (m, 2H), 1.33 – 1.22 (m, 2H), 0.87 (t, J = 7.4 Hz, 3H).

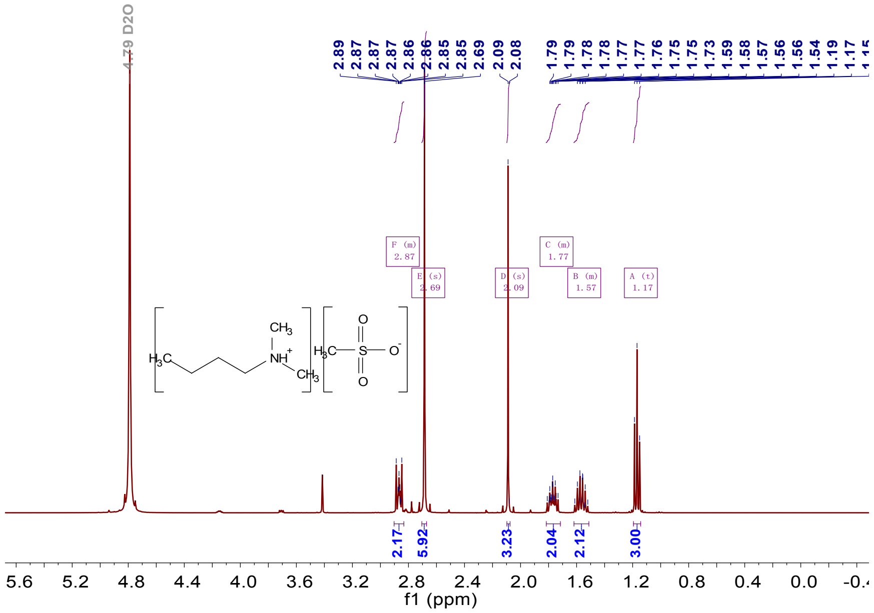

Figure S2.The ^{1}H spectra of [DMBA][$MeSO_3$]

[DMBA][$MeSO_3$] (400 MHz, DMSO-d_6): δ 2.90 – 2.83 (m, 2H), 2.69 (s, 6H), 2.09 (s, 3H), 1.82 – 1.72 (m, 2H), 1.62 – 1.51 (m, 2H), 1.17 (t, J = 7.4 Hz, 3H).

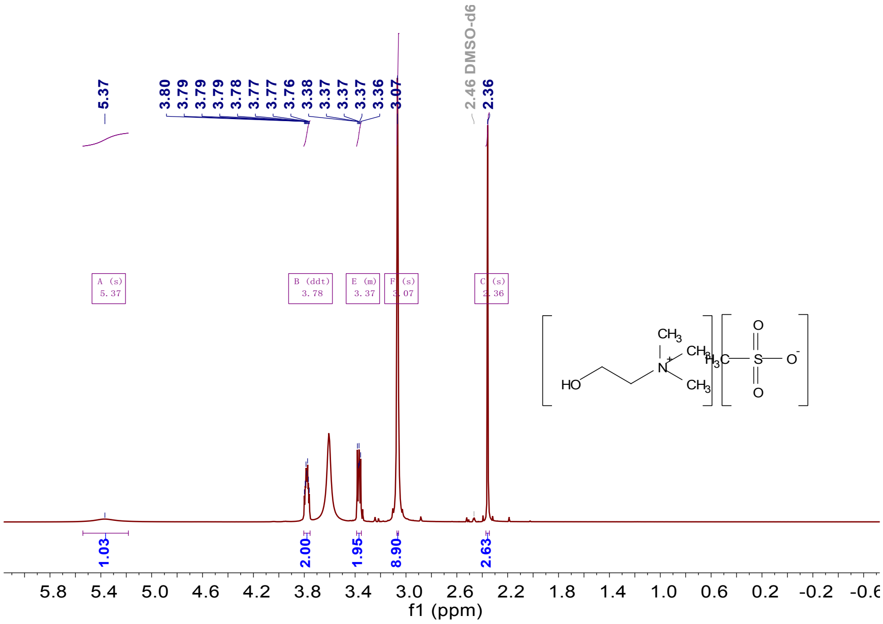

Figure S3. The ^1H spectra of [Ch][$MeSO_3$]

[Ch][$MeSO_3$] (400 MHz, DMSO-d_6): δ 5.37 (s, 1H), 3.78 (ddt, J = 7.9, 5.3, 2.6 Hz, 2H), 3.39 – 3.35 (m, 2H), 3.07 (s, 9H), 2.36 (s, 3H).

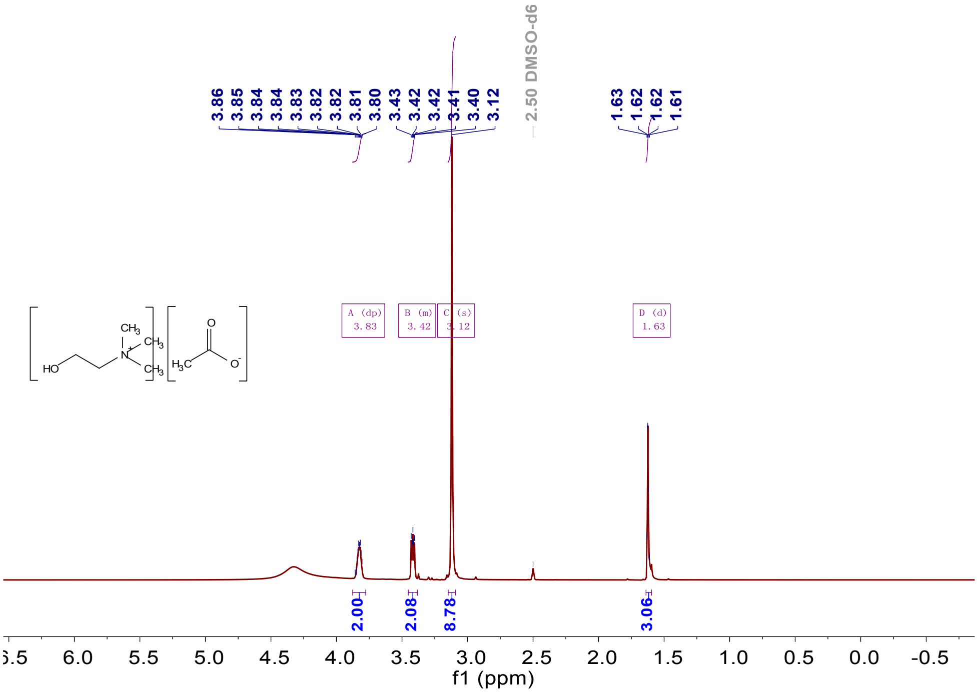

Figure S4. The ^1H spectra of [Ch][OAc]

[Ch][OAc] (400 MHz, DMSO-d_6): δ 3.83 (dp, J = 8.0, 2.8 Hz, 2H), 3.45 – 3.38 (m, 2H), 3.12 (s, 9H), 1.63 (d, J = 2.6 Hz, 3H).

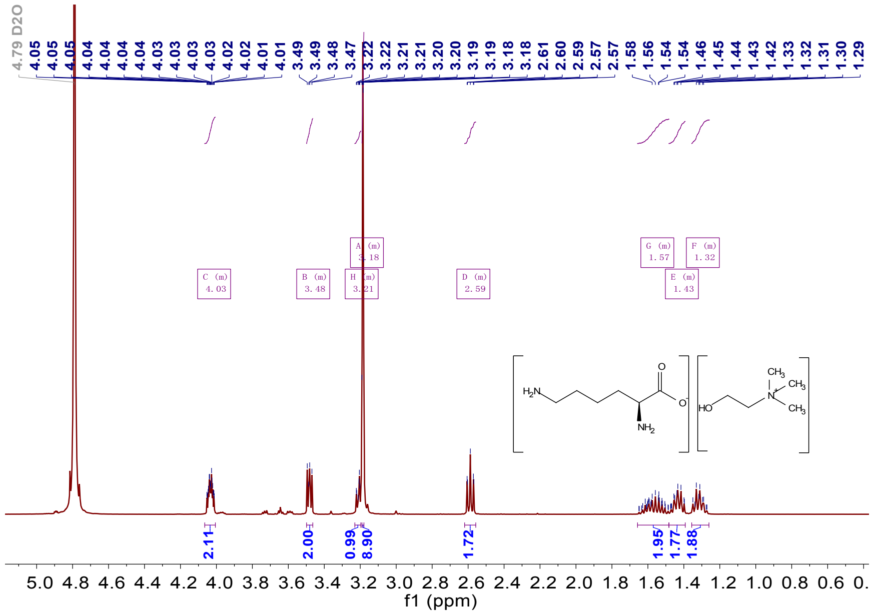

Figure S5. The ^{1}H spectra of [Ch][Lys]

[Ch][Lys] (400 MHz, Deuterium Oxide): δ 4.06 – 4.01 (m, 2H), 3.50 – 3.46 (m, 2H), 3.23 – 3.20 (m, 1H), 3.19 – 3.18 (m, 9H), 2.62 – 2.56 (m, 2H), 1.66 – 1.48 (m, 2H), 1.48 – 1.39 (m, 2H), 1.36 – 1.26 (m, 2H).

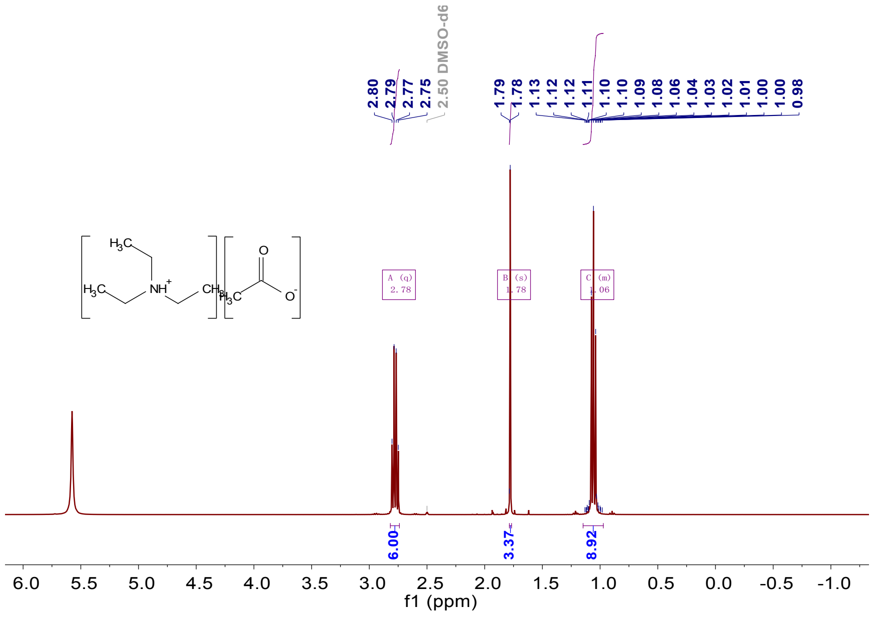

Figure S6. The ^{1}H spectra of [TEA][OAc]

[TEA][OAc] (400 MHz, DMSO-d_6): δ 2.78 (q, J = 7.2 Hz, 6H), 1.78 (s, 3H), 1.15 – 0.97 (m, 9H).

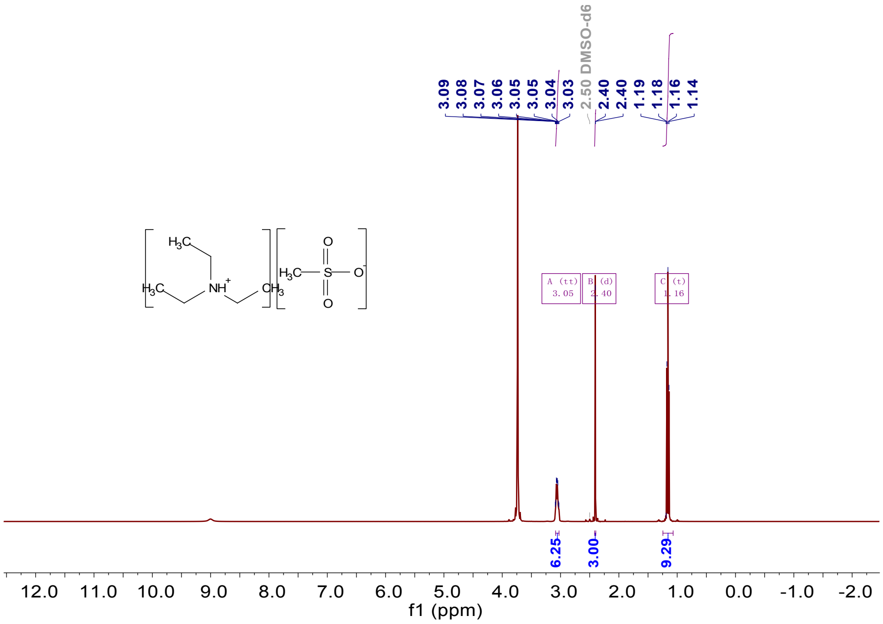

Figure S7. The ^{1}H spectra of [TEA][$MeSO_3$]

[TEA][$MeSO_3$] (400 MHz, DMSO-d_6): δ 3.05 (tt, J = 7.4, 3.6 Hz, 6H), 2.40 (d, J = 0.5 Hz, 3H), 1.16 (t, J = 7.4 Hz, 9H).

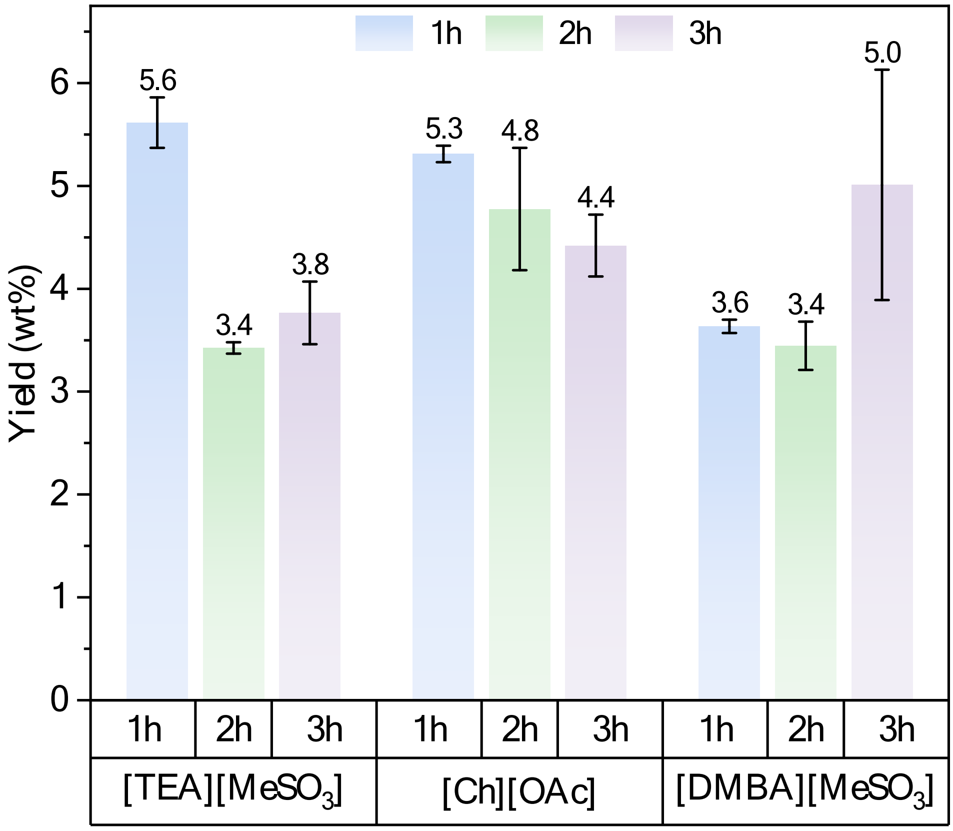

Figure S8. The yield of Pectin from different extraction time.

(Extraction parameters: ethanol as anti-solvents, 10 wt% of solids loading, 30 wt% of water content, 1.0 of ABRs, temperature of 80 °C with the stirring rate of 400 rpm.)

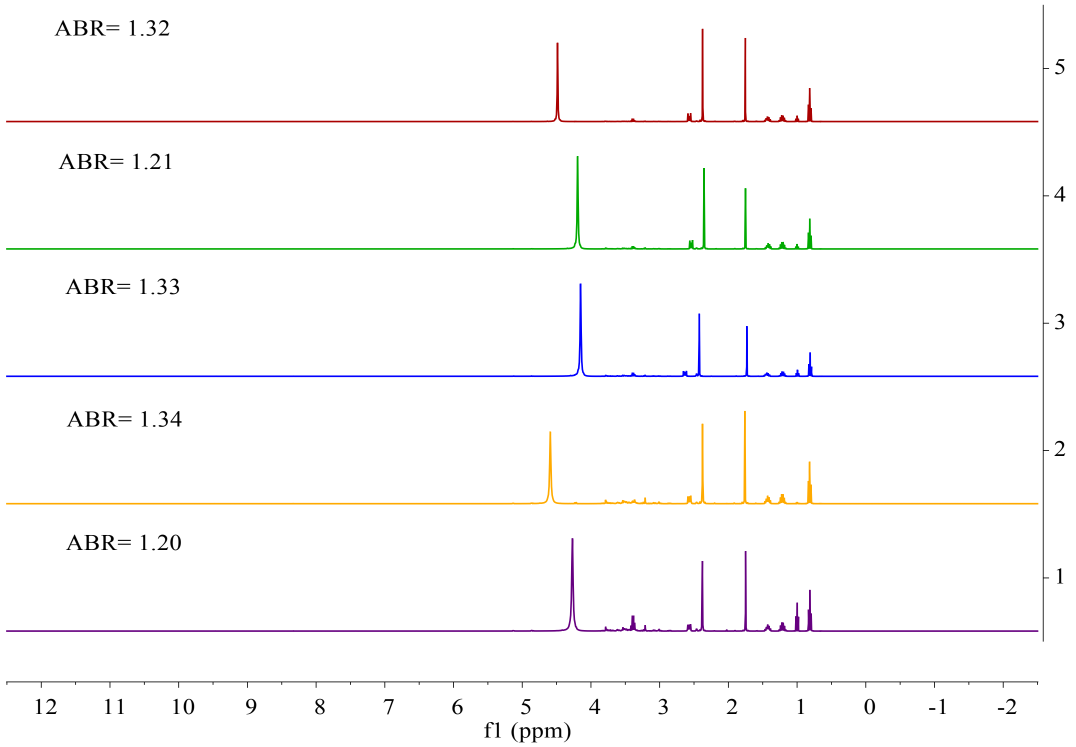

Figure S9. The ^{1}H NMR spectra of recycled [DMBA][OAc]

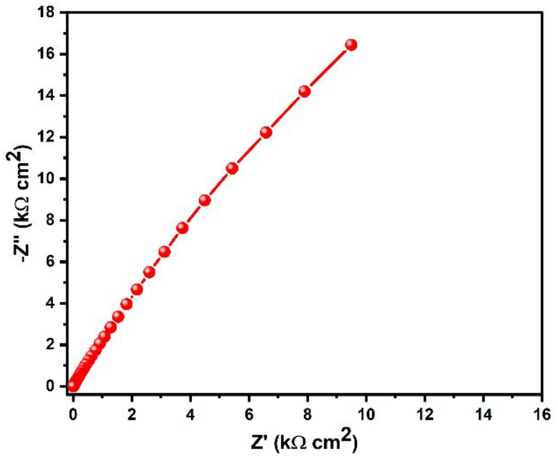

Figure S10. Recorded EIS spectra for ionic conductivity measurement.

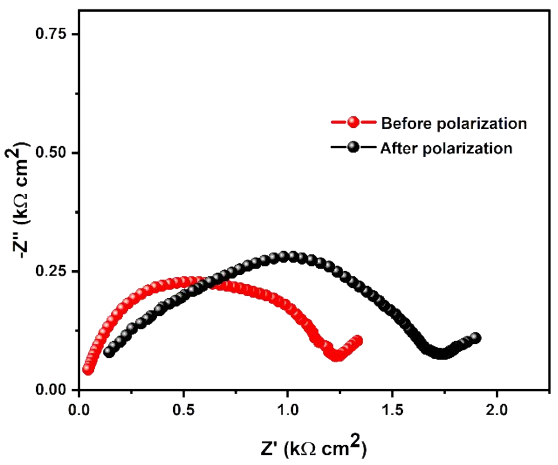

Figure S11. Recorded EIS spectra before and after DC polarization.

Table S1. ANOVA results for a quadratic model of pectin yield

Source	Sum of squares	Df	Mean square	F value	p-value
(2)Temperature	57.76	1	57.76	5776	0.0002
(3)Solid loading	11.22	1	11.22	1122	0.0009
(4)Acid to base ratio	0.30	1	0.30	30	0.0315
1 by 3	0.20	1	0.20	20	0.0460
2 by 3	7.02	1	7.02	702	0.0014
2 by 4	0.30	1	0.30	30	0.0315
3 by 4	0.16	1	0.16	16	0.0572
1*3*4	0.81	1	0.81	81	0.0121
2*3*4	0.49	1	0.49	49	0.0198
Lack of Fit	1.31	7	0.19	19	0.0515
Pure Error	0.02	2	0.01		
Total SS	79.61	18			

Table S2. The concentration of different monosaccharide in the recycled IL (mg/mL)

	sucrose	glucose	fructose
**1st**	262±10	363±14	787±7
**2nd**	519±9	730±22	1581±15
**3rd**	744±13	1027±17	2388±9

Table S3. The element analysis of different part during the pectin extraction process

	C (%)	H (%)	N (%)	O (%)
Apple pomace	39.30	6.61	0.34	53.75
Fresh IL	41.36	12.02	5.72	40.91
Recycled IL	43.67	10.61	4.48	41.25
Pectin	40.71	6.20	2.63	50.47
Solid residue	47.50	6.82	1.78	43.90
Waste liquid after evaporation	42.15	13.41	<0.1	44.44
